# Helium in the adult critical care setting

**DOI:** 10.1186/2110-5820-1-24

**Published:** 2011-07-06

**Authors:** J-L Diehl, V Peigne, E Guérot, C Faisy, L Lecourt, A Mercat

**Affiliations:** 1 Université Paris-Descartes, Paris, France; 2 INSERM U765, Paris, France; 3 Service de Réanimation Médicale, Hôpital Européen Georges Pompidou, AP-HP, Paris, France; 4 Research Department, Air Liquide Santé International, Paris, France; 5 Service de Réanimation Médicale et de Médecine Hyperbare, CHU d'Angers, Angers, France

## Abstract

Helium is a low-density inert gas whose physical properties are very different from those of nitrogen and oxygen. Such properties could be clinically useful in the adult critical care setting, especially in patients with upper to more distal airway obstruction requiring moderate to intermediate levels of FiO_2_. However, despite decades of utilization and reporting, it is still difficult to give any firm clinical recommendation in this setting. Numerous case reports are available in the context of upper airway obstruction of different origins, but there is a lack of controlled studies for this indication. One study reported a helium-induced beneficial effect on surrogates of work of breathing after extubation in non-COPD patients, possibly in relation to laryngeal consequences of tracheal intubation. Physiological benefits of helium-oxygen breathing have been demonstrated in the context of acute severe asthma, but there is a lack of large controlled studies demonstrating an effect on pertinent clinical endpoints, except for a study reported only as an abstract, which mentioned a reduction in the intubation rate in helium-treated patients. Finally, there are a number of physiological studies in the context of COLD-COPD patients demonstrating a beneficial effect, mainly by a reduction in the resistive inspiratory work of breathing but also by a reduction in hyperinflation. Reduction of hypercapnia was mainly observed in spontaneously breathing and noninvasively ventilated helium-treated patients but not in intubated patients during controlled ventilation, suggesting that the decrease in PaCO_2 _was mainly in relation to a diminution in CO_2 _production, related to the diminution in work of breathing and not an improved alveolar ventilation. Moreover, there is little evidence that helium-oxygen could improve parameters of heterogeneity in such patients. Two RCTs were unable to demonstrate a reduction in the intubation rate in such setting, but they were likely underpowered. An adequately powered international multicentric study is ongoing and will help to determinate the exact place of the helium-oxygen mixture in the future. The place of the mixture during the weaning period will deserve further evaluation.

## Introduction

Helium is an inert gas whose physical properties are very different from those of nitrogen and oxygen. Such properties led to consider its use in various medical conditions since the first publication by Barach in the 1930s [1]. The purpose of this review was to detail part of these clinical uses, focusing mainly on obstructive respiratory diseases in the adult critical care setting, and therefore excluding pediatric studies and also studies solely centered on helium-driven nebulization.

### Theoretical and practical considerations

Helium is the second element in the universe, after hydrogen. It is found in very high amounts in the stars. In the earth, helium can be found in very low quantities in the atmosphere and in natural gas fields, or it can be extracted from cleveite, a mineral found in uranium deposits.

The main property of helium--compared with oxygen and nitrogen--is its very low density, as assessed by a volumic mass of 0.18 (in a gaseous state at 0°C) compared with volumic masses of 1.49 and 1.25 for oxygen and nitrogen, respectively. The low density of helium can exert important potential benefits with regards to the airway's dynamic of fluids:

• Mainly, transition of turbulent to laminar flows: as a theoretical example, the density (d) of the mixture is an important determinant of the Reynold's number; which indicates the type of flow in a perfect pipe: *R *= (V.D.d)/η; where V is the mean linear velocity, D is the diameter of the pipe, d is the density of the fluid, and η is the dynamic viscosity of the fluid. When the Reynold's number falls below the 2000 limit, the flow becomes laminar. Because a laminar flow generates much less resistances, use of helium could be of benefit. Inspiratory flow is physiologically turbulent in the upper airways and in the proximal trachea, but in obstructive diseases, such as asthma and COLD/COPD, turbulent flow can be observed more distally in the bronchial tubing.

• Reduction of resistances even in cases of persistent turbulent conditions of flow. Indeed, in cases of turbulent flow, resistances are linearly proportional to the density of the mixture and to the flow value and inversely proportional to the diameter of the pipe at the fifth power.

Another important point to consider, although sometimes neglected, is the 14% higher viscosity of helium compared with nitrogen. In cases of pure laminar flow, such a property directly increases the resistances, as shown by the Poiseuille's law: R = (8.l. η)/Π.r^4 ^(where R is the resistance, l is the length of the pipe, η is the viscosity, and r its radius). This could potentially increase part of the respiratory resistances, because of a larger cross-sectional area flow is mainly laminar in the distal airways, even in obstructive diseases. Moreover, this might have additional clinical implications both for research purpose as well as for care and monitoring, because flow and volume are sometimes measured by methods that need a correcting factor for viscosity (i.e., via pneumotachograph). More generally, useful publications are available that describe the consequences of the use of helium both for mechanical ventilators and for monitoring devices, such as capnometers [2,3].

A third point to consider is the higher thermic conductivity of helium compared with to oxygen and nitrogen. Theoretically, this could favor thermic losses in helium-treated patients. However, to the best of our knowledge, such a complication has never been described in the adult clinical setting and has only been described in children breathing helium-oxygen via a Hood administration system [4]. This also has consequences for some types of monitoring devices.

From all the above-mentioned reasons, one can consider that the low density of the mixture could exert many beneficial effects in cases of airway obstruction:

• mainly by a diminution in the resistive inspiratory work of breathing

• but also by reducing expiratory resistances, leading to less hyperinflation and therefore diminishing elastic inspiratory work of breathing and putting inspiratory muscles in a more favorable geometric configuration

• hypothetically by a redistribution of the gas throughout the lungs, in relation to modifications in time-constants

• and also by reducing arterial tension in CO_2 _through different mechanisms: 1) diminution in CO2 production, via a reduction in the total work of breathing; 2) hypothetically an increase in alveolar ventilation (through a reduction in CO_2 _dead-space facilitated by a better diffusion of CO_2 _in the low-density helium-oxygen mixture; and 3) a reduction in the so-called pendelluft phenomenon, linked to heterogeneous parenchymal viscoelastic properties of the lung

Importantly, the beneficial effects of helium are likely to be observed at low or intermediate FiO_2_. As a consequence, the better candidates for a beneficial effect are patients with upper airways obstruction, severe asthma patients and decompensated COPD or COLD patients, all demonstrating very high respiratory resistances and usually requiring less than 50% FiO_2 _under mechanical ventilation. For each of these situations, we will resume the available data, both from a physiopathological point of view but also with consideration of major clinical endpoints.

### Upper airway obstruction

Upper airway obstruction, especially if transient, is probably one of the better potential indications of helium-oxygen breathing. Some case reports are available that describe a beneficial use of the mixture in spontaneously breathing patients, mainly in the pediatric literature but also in adult patients: the potential indications are malignancies [5], bilateral vocal cord dysfunction complicating short-term intubation [6], or radiation therapy [7,8]. Unfortunately, available data are generally limited to the description of clinical courses in small numbers of patients, without helium-free controlled periods and/or measurement of pertinent physiological parameters, such as the work of breathing or airway resistances.

Interestingly, Jaber and coworkers performed a physiological study during the postextubation period that compared short periods of air-breathing and helium-oxygen breathing in 18 non-COPD patients [9]. They mainly found a helium-associated reduction in the transdiaphragmatic pressure inspiratory swings and in the pressure-time index (these two parameters are surrogates of the work of breathing) as well as an improvement in the comfort score of the patients. Such a benefit could be in relation to a beneficial effect of helium-oxygen breathing on clinically latent postintubation upper airway obstruction related to laryngeal injuries [10].

### Asthma

Numerous case studies have been published in the setting of acute severe asthma, reporting favorable outcomes. Unfortunately, no firm conclusion can be drawn from such cases with regard to the interest to add helium-oxygen breathing, systematically or as a rescue therapy, to the standard treatment of acute severe asthma. More rigorously, Manthous et al. studied in the emergency setting 27 patients with acute asthma exacerbation who received standard treatment (beta-agonist aerosols and intravenously administered methylprednisolone); 16 patients were allocated to breathe helium-oxygen, whereas the remaining 11 patients acted as a control group [11]. The authors documented in the helium-oxygen group a more marked fall in the pulsus paradoxus and a greater improvement in the peak expiratory flow (measured via a helium-oxygen calibrated peak flow meter) than in the control group. In another study, 23 patients with acute severe asthma were randomized in the emergency department to breathe air-oxygen or helium-oxygen in addition to standard treatment [12]. The authors documented a more rapid improvement during the first hour (as assessed by the peak-flow and the dyspnea score) in the helium-oxygen group, without clinically significant differences between the two groups beyond this time-point. Finally, Sattonnet et al. performed a randomized, multicenter, controlled study in the prehospital setting in 203 patients with acute severe asthma who needed hospitalization in the ICU [13]. The authors reported in an abstract a significant difference in the intubation rate in favor of helium-oxygen: 7% versus 1%. They also reported a diminution in the ICU length of stay compared with the control group.

Altogether, there is to date no firm argument in favor of systematic administration of helium-oxygen therapy for acute severe asthma, as confirmed by a meta-analysis of ten randomized, controlled trials (seven involving adult patients), including 544 acute asthma patients [14]. The helium-oxygen mixture could be of value in the more severe patients, who might be at risk of intubation, while awaiting the effects of standard therapy. However, the level of evidence seems too low to recommend firmly its administration in this setting. Interestingly, no data are available regarding the use of a combination of helium-oxygen and noninvasive ventilation in such a situation.

### COLD and COPD

The available amount of clinically relevant data is higher in COPD/COLD exacerbations than in the two previous situations, with well-performed physiological studies but also with published RCTs assessing pertinent clinical endpoints. We will discuss three different situations: 1) use of helium during spontaneous breathing and/or noninvasive ventilation in cases of acute exacerbation; 2) use of helium during invasive ventilation; and 3) use of helium during the weaning period.

### Use of helium during spontaneous breathing and/or noninvasive ventilation in cases of acute exacerbation

Gerbeaux et al. reported provocative results obtained in 81 decompensated COPD patients in the emergency setting [15]. Forty-two patients received standard treatment, whereas helium-oxygen during spontaneous breathing was administered in the 39 remaining patients. Unfortunately, the study was observational and the administration of helium-oxygen was not randomized but kept at the discretion of the physician in charge. However, no significant difference was found at admission between the two groups with regards to any of the relevant clinical parameters. The authors reported impressive results with regards to intubation rates (50% in the control group vs. 8% in helium-oxygen group) compared with the mortality rates (24% and 3% respectively) and finally with regards to the ICU and hospital lengths of stay for survivors. Unfortunately, the nonrandomized design of this monocentric study precludes any firm clinical recommendation in favor of the use of helium-oxygen breathing in such a situation. To date, no confirmation study of these results is available.

Jolliet et al. evaluated the interest of helium-oxygen in combination with pressure-support noninvasive ventilation in 19 decompensated COPD patients [16]. They compared three different situations: air-oxygen while spontaneously breathing, air-oxygen pressure support, and helium-oxygen pressure support. They reported beneficial effects of helium-oxygen pressure support not only in comparison with the spontaneously breathing periods but also in comparison with the conventional pressure support period, mainly with regard to the dyspnea Borg scale.

Jaber et al. extended these data in a comparable setting while studying ten COPD patients at two different levels of pressure support during noninvasive ventilation [17]. They mainly reported a helium-oxygen-associated diminution in the inspiratory work of breathing compared with air-oxygen periods. They also reported a beneficial effect in terms of PaCO_2 _decrease during the helium-oxygen periods.

Finally, two multicenter RCTs were performed aiming to demonstrate a reduction in the intubation rate in exacerbation of COPD/COLD patients [18,19]. Jolliet et al. included 123 COPD patients and Maggiore et al. included 204 COLD patients. The intubation rates were 20.3% and 13.5% in the air and helium-oxygen groups, respectively, in the study by Jolliet et al. The intubation rates were 30.4% and 24.5%, respectively, in the study by Maggiore et al. These differences were not statistically significant. As secondary endpoints, the two groups of investigators reported a beneficial effect of helium-oxygen in term of improvements of pH and PaCO_2 _values. Jolliet et al. also performed a cost analysis, which favored the use of helium-oxygen, mainly because a shorter duration of mechanical ventilation and a shorter ICU and hospital length of stay. Finally, it is likely that the two studies were underpowered to demonstrate a difference in the principal endpoint.

### Use of helium during invasive ventilation

Tassaux et al. performed a study in 23 decompensated intubated COPD patients that compared two periods of air-oxygen ventilation to one period of helium-oxygen ventilation; all other parameters remained unchanged [20]. They mainly found helium-associated significant reductions in Pmax, static intrinsic PEEP (staticPEEPi), and trapped volume. There were no differences in oxygenation values, CO_2 _value, or any hemodynamic parameter. Gainnier et al. performed a study in 23 intubated COPD patients that compared two periods of air-oxygen and helium-oxygen [21]. They reported a decrease in work of breathing, implying its resistive and elastic components as a decrease in staticPEEPi.

These data were extended by a study of our group including 13 COPD patients [22]. We used a similar design than in the study by Tassaux [20]. We similarly found significant reductions in Pmax and in staticPEEPi, although the latest was less pronounced than in the study by Jolliet et al. (Table 1). Accordingly, we found no differences in oxygenation values, PaCO_2 _values, as well as in physiological dead-space. Finally, we took the opportunity to test the hypothesis that helium-oxygen could improve the heterogeneity of ventilation in such patients. As previously reported in similar patients, we observed during air-oxygen ventilation major differences between staticPEEPi and dynamic intrinsic PEEP (dynPEEPi) [23]. Interestingly, helium induced a small diminution in each of these parameters, whereas the dynPEEPi/staticPEEPi ratio remained unchanged. The dynPEEPi/staticPEEPi ratio is thought to be related to regional differences in mechanical properties within the lung--a phenomenon often referred as pendelluft--and/or viscoelastic pressure losses [22]. To date, there is no useful clinical way to appreciate separately the absolute magnitude and the relative contribution of each of these two factors, but one can reasonably consider that helium-oxygen had little influence on this global index of heterogeneity. Supporting this interpretation is the fact that we also observed no significant change in the difference in pressure between the pressure (P_1_) observed during an inspiratory pause after the rapid initial fall in pressure, at the first point of zero flow, and the pressure observed after 5 seconds of occlusion (Ppl). As for the dynPEEPi/staticPEEPi ratio, (P_1 _- Ppl) can be attributed both to pendelluft and stress relaxation.

**Table 1:** Results obtained during three periods of 1 hour of controlled mechanical ventilation in 13 COPD patients during the first 24 after intubation

	First period air-oxygen	Second period helium-oxygen	Third period air-oxygen
Pmax, cmH_2_O	35.4 ± 6.7	31.6 ± 7.6*	34.9 ± 6.9
Ppl, cmH2O	22.3 ± 4.9	20.9 ± 5.5*	21.7 ± 4.7
P_1 _- Ppl, cmH_2_O	4.5 ± 2.2	4.7 ± 1.7	4.6 ± 2.3
statPEEPi, cmH_2_O	11.7 ± 4.0	10.3 ± 4.3*	10.9 ± 3.8
dynPEEPi, cmH_2_O	3.1 ± 1.0	2.7 ± 0.7*	3.0 ± 0.8
PaO_2_, mmHg	77 ± 12	79 ± 15	80 ± 12
PaCO_2_, mmHg	59 ± 12	62 ± 14	61 ± 14
VD/VT, %	71 ± 6	69 ± 5	69 ± 7

Ppl = plateau pressure measured after an endinspiratory pause of 5 sec; P_1 _- Ppl = difference between the pressure (P_1_) measured after the rapid initial fall in pressure during an inspiratory pause, at the first point of zero flow, and Ppl; statPEEPi = static intrinsic PEEP; dynPEEPi = dynamic intrinsic PEEP; VD/VT = physiological dead-space

Results are expressed as mean ± SD

*Indicates a significant difference at the 0.05 level.

Finally, Tassaux et al. studied ten recovering intubated COPD patients while ventilated with pressure support [24]. They consistently reported a helium-induced reduction in PEEPi, in the number of ineffective breaths, in inspiratory effort, and in work of breathing for a given level of pressure support.

### Use of helium during the weaning period

We performed a study in 13 severe intubated COPD patients at the end of the weaning process, measuring ventilatory variables and work of breathing just before extubation during two periods of air-oxygen and helium-oxygen [25]. Repetition of the measurements was possible in five patients after extubation. Before extubation, we observed mainly a diminution in the total work of breathing as well as in its resistive component. We also found a significant decrease in PEEPi during helium-oxygen breathing. Similar results were observed after extubation in the five patients who tolerated the repetition of measurements. Interestingly, we found that the absolute reduction in work of breathing was correlated with the basal value during air-oxygen breathing. We also observed that one of the patients exhibiting the lowest levels of WOB during air/oxygen breathing experienced an increase in WOB with the helium-oxygen mixture. This was perhaps in relation to a predominantly distal airway obstruction with a deleterious effect of the mixture possibly in relation with its higher viscosity compared with air-oxygen. Altogether, these results emphasized the need to search for predictors useful to identify responders to helium-oxygen breathing, because the physiological response could be variable for a patient to another, with possibility of detrimental effects.

## Conclusions and perspectives

Despite decades of reports and utilization, the exact place of helium-oxygen in the adult critical care setting is still not well-defined, although many studies suggest a potential benefit in upper airway obstruction, in severe asthma, and in exacerbations of COPD/COLD. The main body of evidence is found in COPD-COLD patients, with not only case reports or uncontrolled small series but also a consequent number of rigorous physiological study and RCTs. In the future, it will be important to consider the results of an international ongoing adequately powered RCT designed to evaluate helium-oxygen not only in combination with noninvasive ventilation, but also during the noninvasive free periods of spontaneous breathing (ClinicalTrials.gov identifier: NCT01155310). It also will be important to consider further evaluation of the clinical benefit of the mixture in the weaning period.

## Competing interests

Dr. Lecourt has been employed at Air Liquide Santé. The remaining authors have not disclosed any potential conflicts of interest.

## Authors' contributions

JLD was responsible for literature research, manuscript writing and final approval. VP, EG, CF, LL and AM were responsible for final approval.
